# Evidence of intrauterine device insertion by nurses in Primary Health Care: an integrative review

**DOI:** 10.1590/0034-7167-2023-0134

**Published:** 2024-02-26

**Authors:** Lays Souza de Oliveira, Patrícia Madalena Vieira Hermida, Elizimara Ferreira Siqueira, Juliana Cipriano Braga Silva de Arma, Larissa Scheeren Thomas, Indiara Sartori Dalmolin

**Affiliations:** I Prefeitura Municipal de Florianópolis. Florianópolis, Santa Catarina, Brazil

**Keywords:** Intrauterine Devices, Nurses, Primary Health Care, Advanced Practice Nursing, Long-Acting Reversible Contraception, Dispositivos Intrauterinos, Enfermeros, Atención Primaria de Salud, Enfermería de Práctica Avanzada, Anticoncepción Reversible de Larga Duración, Dispositivos Intrauterinos, Enfermeiros, Atenção Primária à, Saúde, Prática Avançada de Enfermagem, Contracepção Reversível de Longo Prazo

## Abstract

**Objectives:**

to synthesize and analyze evidence on intrauterine device insertion by nurses in Primary Health Care.

**Methods:**

an integrative review, carried out in the BDENF, CINAHL, LILACS, SciELO, Scopus, PubMed and Web of Science databases in June 2022, delimiting the period from 1960 to 2022.

**Results:**

141 articles were identified in the initial search, and 10 studies made up the final sample. Four (40%) were developed in the United States and one (10%) in Brazil, with publications from 1979 to 2021. The findings were grouped into three categories: Nurse training to insert an intrauterine device; Nurses’ competency to insert an intrauterine device; and Women’s access to intrauterine devices.

**Conclusions:**

nurse theoretical and practical training is a prominent element, consolidated in the favorable outcomes of insertions performed by nurses and satisfaction among women, a practice that has expanded access to the contraceptive method in Primary Health Care.

## RESUMO

**Objetivos:**

sintetizar e analisar as evidências da inserção de dispositivo intrauterino por enfermeiros na Atenção Primária à Saúde.

**Métodos:**

revisão integrativa, realizada nas bases de dados BDENF, CINAHL, LILACS, SciELO, Scopus, PubMed e *Web of Science* em junho de 2022, delimitando-se o período de 1960 a 2022.

**Resultados:**

identificaram-se 141 artigos na busca inicial, e 10 publicações compuseram a amostra final. Quatro (40%) foram desenvolvidos nos Estados Unidos e um (10%) no Brasil, sendo publicações de 1979 a 2021. Os achados foram agrupados em três categorias: Treinamento dos enfermeiros para inserção de dispositivo intrauterino; Competência dos enfermeiros para inserção de dispositivo intrauterino; e Acesso das mulheres aos dispositivos intrauterinos.

**Conclusões:**

o treinamento teórico e prático dos enfermeiros é um elemento de destaque, consolidado nos desfechos favoráveis das inserções realizadas por enfermeiros e satisfação entre as mulheres, prática que tem ampliado o acesso ao método contraceptivo na Atenção Primária à Saúde.

## RESUMEN

**Objetivos:**

sintetizar y analizar la evidencia sobre la inserción de dispositivos intrauterinos por parte de enfermeras en la Atención Primaria de Salud.

**Métodos:**

revisión integrativa, realizada en las bases de datos BDENF, CINAHL, LILACS, SciELO, Scopus, PubMed y Web of Science en junio de 2022, delimitando el período de 1960 a 2022.

**Resultados:**

se identificaron 141 artículos en la búsqueda inicial y 10 publicaciones conformaron la muestra final. Cuatro (40%) fueron desarrollados en Estados Unidos y uno (10%) en Brasil, con publicaciones de 1979 a 2021. Los hallazgos se agruparon en tres categorías: Capacitación de enfermeras para insertar un dispositivo intrauterino; Competencia de las enfermeras para insertar un dispositivo intrauterino; y Acceso de las mujeres a los dispositivos intrauterinos.

**Conclusiones:**

la formación teórica y práctica de los enfermeros es un elemento destacado, consolidado en los resultados favorables de las inserciones realizadas por los enfermeros y la satisfacción de las mujeres, práctica que ha ampliado el acceso al método anticonceptivo en la Atención Primaria de Salud.

## INTRODUCTION

Long-acting reversible contraception (LARC) has been motivating new public health policies worldwide in an attempt to overcome the rates of unwanted pregnancies, abortions and maternal mortality^(1)^, highlighting, among them, the intrauterine device (IUD), object of this study.

The IUD, made of solid material, usually in the shape of a T, is placed in the uterine cavity and aims to prevent pregnancy. It is available in different configurations, the main ones being the copper IUD and the levonorgestrel-releasing intrauterine system (LNG-IUS). In Brazil, the Ministry of Health (MoH) makes copper IUD available free of charge through the Brazilian Health System (SUS - *Sistema Único de Saúde*)^(2)^.

From this perspective, the eligibility criteria for the use of each contraceptive method must be considered. The copper IUD and the hormonal IUD can be offered to most women, nulliparous and multiparous, after vaginal or cesarean birth, after abortion in the first trimester, for adolescents and women in perimenopause. For those who have contraindications to hormonal methods, the copper IUD is an option^(3-5)^.

IUD are advantageous because they are easily reversible, have low pregnancy rates, few unwanted effects, do not depend on frequent maintenance users’ behaviors and have local (intrauterine) action. Specifically about the copper IUD, it is more cost-effective, effective for up to 10 years, and can be used as an emergency contraceptive, having no effect on lactation or sexual function^(3,6)^. Furthermore, its mechanism of action does not use hormones, an advantage that, in most cases, is the main criterion for choice among women^(7)^. LNG-IUS has the specific advantages of being effective for up to five years, reducing menstrual volume and colic, and can be used as a treatment for excessive bleeding^(5)^.

Despite the advantages, LARC are underused around the world. According to a United Nations study^(8)^, the estimated prevalence of IUD use among women of reproductive age (15-49 years) in 2019 was 8.4% worldwide, with 10.7% in Asia, 8.1% in Europe, 7.6% in America North, 4.6% in Latin America and the Caribbean, 3.4% in Oceania and 2.6% in Africa. Among the barriers to using the method, organizational, such as low availability of the method or high number of criteria for its insertion, lack of institutional protocols, restriction of nurses’ performance or limitation in the training of professionals, and individual, especially the low level of knowledge about the method, stand out^(1,9)^.

Evidence shows that nurses have a relevant role in IUD insertion in different locations around the world, such as Australia^(10)^, the United States^(11-12)^, England^(13)^, India^(14)^ and Brazil^(15)^. In the country, nurses have had their practice regulated by the Federal Nursing Council (COFEN - *Conselho Federal de Enfermagem*) since 2010, which states that this professional has the capacity and legal competency to insert and remove the IUD after qualification and training^(16)^. In 2022, a new resolution began to standardize this practice of nurses, providing for their competencies regarding device insertion, review and removal, including detailing training time and the places where they work for reproductive planning actions, such as in Primary Health Care (PHC)^(17)^.

PHC constitutes an important setting for offering IUD, as it is the main gateway into health systems, highlighting the importance of nurses in planning and reproductive health actions in this care space, through guidance on contraceptive methods, contributing to expanding women’s access to IUD^(1,6)^.

Considering the above, considering that unwanted pregnancy remains a public health problem and that regulation of IUD insertion practice by nurses in Brazil is an object of constant discussion, this study is justified by the possibility of knowing the scientific evidence of device insertion by PHC nurses worldwide, which could contribute to strengthening this advanced practice of nurses in this care scenario for its legal support and institutional consolidation as well as expanding access to IUD through nurses’ actions so that new research on the topic can be envisioned.

## OBJECTIVES

To synthesize and analyze evidence of IUD insertion by nurses in PHC.

## METHODS

This is an integrative literature review, which followed the steps suggested by Ganong^(18)^ (1987): 1) review objective formulation and guiding question selection; 2) determination of inclusion and exclusion criteria for the literature search; 3) search and selection of primary studies, arranging them in tables and organizing common ideas; 4) critical analysis of included studies; 5) data discussion and interpretation; and 6) clear and complete review presentation.

In the first stage, the guiding question of this review was defined as: what is the evidence on IUD insertion by nurses in PHC? Then, the eligibility criteria for the studies were defined, including original research, experience reports, case and review studies, with a quantitative or qualitative approach, published from 1960 to 2022, in an indexed journal, in article format and in Portuguese, English or Spanish, and which had PHC nurses as participants, even if not exclusively. Exclusion criteria included letters, editorials, theses, dissertations, monographs, books, works unavailable in full electronically or in print and those not related to the topic of study or that do not answer the research question, in addition to duplicate studies in the databases. The research time frame is justified by the fact that scientific literature records the first IUD insertions by nurses in the world in the 1960s^(19)^.

The searches were conducted in June 2022, using advanced search tool in the databases: Nursing Database (BDENF); Cumulative Index to Nursing and Allied Health Literature (CINAHL); Embase; Latin American and Caribbean Literature in Health Sciences (LILACS); Scientific Electronic Library Online (SciELO); Scopus; U.S. National Library of Medicine (PubMed); and Web of Science (WOS) via Virtual Private Network (VPN) from the *Universidade Federal de Santa Catarina* (UFSC).

To define the descriptors, the terms systematized in the Health Sciences Descriptors (DeCS) and the Medical Subject Headings (MeSH) were used. Different combinations of Boolean operators “AND” and “OR” were used to identify the largest number of articles possible. The search strategy was adapted for each database according to its specificities, which was carried out by the researcher together with a librarian from UFSC (Chart 1).

**Chart 1:** Databases with their respective search strategies

Databases and search strategies
**BDENF and LILACS** (“Intrauterine Devices” OR “Contraceptive IUD” OR “Contraceptive IUDs” OR “Intrauterine Contraceptive Device” OR “Intrauterine Contraceptive Devices” OR “Intrauterine Device” OR “Unmedicated IUD” OR “Unmedicated IUDs” OR “*Dispositivos Intrauterinos*” OR “*Anticoncepcionais Intrauterinos*” OR “*Dispositivo Intrauterino*” OR “*Anticonceptivos Intrauterinos*” OR “*Contraceptivos Intrauterinos*”) AND (“Nursing Care” OR “Nursing” OR “Nursings” OR “Nurses” OR “Nurse” OR “*Cuidados de Enfermagem*” OR “*Enfermagem*” OR enfermeir^*^ OR “*Atención de Enfermería*” OR “*enfermeria*” OR enfermer^*^) AND (“Primary Health Care” OR “Primary Healthcare” OR “Primary Care” OR “basic health care” OR “basic care” OR “basic service” OR “Primary Care Nursing” OR “*Atenção Primária à Saúde*” OR “*Atenção Básica*” OR “*Atenção Primária*” OR “*Atendimento Básico*” OR “*Atendimento Primário*” OR “*Cuidados de Saúde Primários*” OR “*Cuidado de Saúde Primário*” OR “*Cuidados Primários*” OR “*Cuidado Primário*” OR “*Cuidado de Saúde Básico*” OR “*Cuidados de Saúde Básicos*” OR “*Cuidado Básico*” OR “*Cuidados Básicos*” OR “*Enfermagem de Atenção Primária*” OR “*Atención Primaria de Salud*” OR “*Atención Primaria*” OR “*Atención Básica*” OR “*Cuidado de la Salud Primarios*” OR “*Cuidados Primarios*” OR “*servicios básicos de salud*” OR “*servicio básico*” OR “*servicios básicos*” OR “*cuidado básico de salud*” OR “*cuidados básicos de salud*” OR “*Enfermería de Atención Primaria*”)
**CINAHL** (“Intrauterine Devices” OR “Contraceptive IUD” OR “Contraceptive IUDs” OR “Intrauterine Contraceptive Device” OR “Intrauterine Contraceptive Devices” OR “Intrauterine Device” OR “Unmedicated IUD” OR “Unmedicated IUDs”) AND (“Nursing Care” OR “Nursing” OR “Nursings” OR “Nurses” OR “Nurse”) AND (“Primary Health Care” OR “Primary Healthcare” OR “Primary Care” OR “basic health care” OR “basic care” OR “basic service” OR “Primary Care Nursing”)
**EMBASE** (“Intrauterine Devices” OR “Contraceptive IUD” OR “Contraceptive IUDs” OR “Intrauterine Contraceptive Device” OR “Intrauterine Contraceptive Devices” OR “Intrauterine Device” OR “Unmedicated IUD” OR “Unmedicated IUDs”) AND (“Nursing Care” OR “Nursing” OR “Nursings” OR “Nurses” OR “Nurse”) AND (“Primary Health Care” OR “Primary Healthcare” OR “Primary Care” OR “basic health care” OR “basic care” OR “basic service” OR “Primary Care Nursing”
**SciELO** (“Intrauterine Devices” OR “Contraceptive IUD” OR “Contraceptive IUDs” OR “Intrauterine Contraceptive Device” OR “Intrauterine Contraceptive Devices” OR “Intrauterine Device” OR “Unmedicated IUD” OR “Unmedicated IUDs” OR “*Dispositivos Intrauterinos*” OR “*Anticoncepcionais Intrauterinos*” OR “*Dispositivo Intrauterino*” OR “*Anticonceptivos Intrauterinos*” OR “*Contraceptivos Intrauterinos*”) AND (“Nursing Care” OR “Nursing” OR “Nursings” OR “Nurses” OR “Nurse” OR “*Cuidados de Enfermagem*” OR “*Enfermagem*” OR enfermeir^*^ OR “*Atención de Enfermería*” OR “*enfermeria*” OR enfermer^*^) AND (“Primary Health Care” OR “Primary Healthcare” OR “Primary Care” OR “basic health care” OR “basic care” OR “basic service” OR “Primary Care Nursing” OR “*Atenção Primária à Saúde*” OR “*Atenção Básica*” OR “*Atenção Primária*” OR “*Atendimento Básico*” OR “*Atendimento Primário*” OR “*Cuidados de Saúde Primários*” OR “*Cuidado de Saúde Primário*” OR “*Cuidados Primários*” OR “*Cuidado Primário*” OR “*Cuidado de Saúde Básico*” OR “*Cuidados de Saúde Básicos*” OR “*Cuidado Básico*” OR “*Cuidados Básicos*” OR “*Enfermagem de Atenção Primária*” OR “*Atención Primaria de Salud*” OR “*Atención Primaria*” OR “*Atención Básica*” OR “*Cuidado de la Salud Primarios*” OR “*Cuidados Primarios*” OR “*servicios básicos de salud*” OR “*servicio básico*” OR “*servicios básicos*” OR “*cuidado básico de salud*” OR “*cuidados básicos de salud*” OR “*Enfermería de Atención Primaria*”)
**Scopus** (“Intrauterine Devices” OR “Contraceptive IUD” OR “Contraceptive IUDs” OR “Intrauterine Contraceptive Device” OR “Intrauterine Contraceptive Devices” OR “Intrauterine Device” OR “Unmedicated IUD” OR “Unmedicated IUDs”) AND (“Nursing Care” OR “Nursing” OR “Nursings” OR “Nurses” OR “Nurse”) AND (“Primary Health Care” OR “Primary Healthcare” OR “Primary Care” OR “basic health care” OR “basic care” OR “basic service” OR “Primary Care Nursing”)
**PubMed** (“Intrauterine Devices”[Mesh] OR “Intrauterine Devices” OR “Contraceptive IUD” OR “Contraceptive IUDs” OR “Intrauterine Contraceptive Device” OR “Intrauterine Contraceptive Devices” OR “Intrauterine Device” OR “Unmedicated IUD” OR “Unmedicated IUDs” OR “Intrauterine Devices, Copper”[Mesh]) AND (“Nursing Care”[Mesh] OR “Nursing Care” OR “Nursing”[Mesh] OR “Nursing” OR “Nursings” OR “Nurses”[Mesh] OR “Nurses” OR “Nurse”) AND (“Primary Health Care”[Mesh] OR “Primary Health Care” OR “Primary Healthcare” OR “Primary Care” OR “basic health care” OR “basic care” OR “basic service” OR “Primary Care Nursing” OR “Primary Care Nursing”[Mesh])
**Web of Science** (“Intrauterine Devices” OR “Contraceptive IUD” OR “Contraceptive IUDs” OR “Intrauterine Contraceptive Device” OR “Intrauterine Contraceptive Devices” OR “Intrauterine Device” OR “Unmedicated IUD” OR “Unmedicated IUDs”) AND (“Nursing Care” OR “Nursing” OR “Nursings” OR “Nurses” OR “Nurse”) AND (“Primary Health Care” OR “Primary Healthcare” OR “Primary Care” OR “basic health care” OR “basic care” OR “basic service” OR “Primary Care Nursing”)

After searching the databases, all citations found were exported to the Rayyan - Intelligent Systematic Review web application, developed by the Qatar Computing Research Institute (QCRI), to assist in study selection and organization. Rayyan QCRI consists of a tool that helps in exploring and filtering searches for studies eligible for systematic reviews, mainly in the title and abstract screening phase. It allows assessing studies with the blinding of the assistant reviewer, which contributes to reliability in information selection and methodological accuracy^(20)^. Subsequently, duplicate studies were excluded.

**Figure 1:**
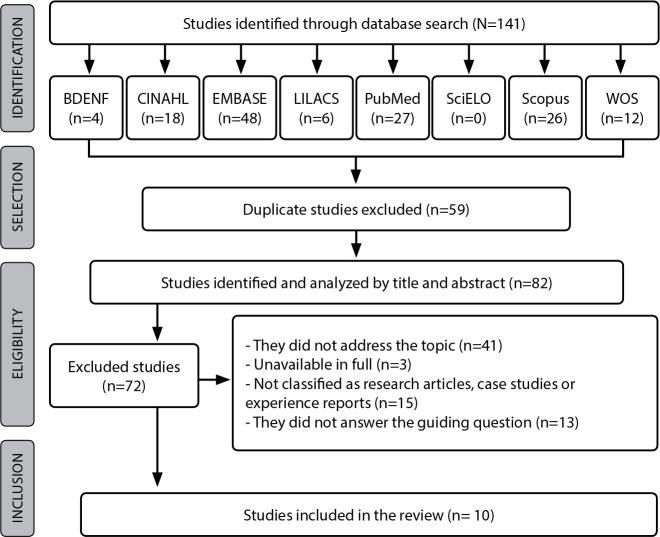
Flowchart for identification and selection of studies, prepared based on PRISMA recommendation

To select the articles that constituted the sample, two independent researchers read the titles and abstracts. From this, both read the studies in full, which were selected based on inclusion and exclusion criteria. It is worth noting that the studies that presented decision-making conflicts in the selection process were analyzed by a third researcher. Furthermore, the Statement for Reporting Systematic Review and Meta-Analyses of Studies (PRISMA) checklist recommendations were used to select studies^(21)^, as shown in Figure 1.

To organize and analyze the data, the researcher created a spreadsheet in Google Spreadsheets^®^ with the data: title; year of publication; country where the research was carried out; study design; objective; participants; main results; conclusion; and level of evidence. Each study received an identification code composed of the letter “S” followed by an Arabic number (S1, S2…).

For critical analysis of results, interpretation of findings and discussion, the themes that emerged were subdivided into three categories: Nurse training to insert an intrauterine device; Nurses’ competency to insert an intrauterine device; and Women’s access to intrauterine devices.

Regarding the level of scientific evidence of the studies, the classification into seven levels was applied: level I, evidence from a systematic review or meta-analysis of all relevant randomized controlled clinical trials (RCTs); level II, evidence from controlled RCTs with good design; level III, evidence derived from well-designed clinical trials without randomization; level IV, evidence obtained from well-designed cohort and case-control studies; level V, evidence resulting from a systematic review of descriptive and qualitative studies; level VI, evidence from a single descriptive or qualitative study; level VII, evidence originating from authority opinion and/or reports from expert committees^(22)^.

The present study, as it is an integrative literature review, did not require approval from the Research Ethics Committee, however all ethical aspects provided for in Law 9,610/98 on copyright were considered, such as citing the authors of selected articles and preserving the ideas, concepts and findings presented in the investigations^(23)^.

## RESULTS

Initially, a total of 141 studies were identified, of which 10 constituted the final sample of the present review. Of this sample quantity, four (40%) studies were developed in the United States, three (30%) in England, one (10%) in Burkina Faso, one (10%) in the Philippines, and one (10%) in Brazil, which was carried out in the city of Florianópolis, Santa Catarina. The temporal variation between the first and last publication was 42 years (1979 - 2021), with only three (30%) published in the last five years (Chart 2).

**Chart 2:** Characteristics of studies that made up the review sample, Florianópolis, Santa Catarina, Brazil, 2022

Code	Title	Year/country of origin	Study design/LoE	Participants
S1^(24)^	The Bohol IUD program	1979/Philippines	Experience report/VII	Nurses^*^ (n=40) and midwives (n=40)
S2^(25)^	The effectiveness of non-physicians as providers of family planning services	1979/United States	Literature review/VII	Not applicable
S3^(26)^	Managing change in primary care: part 1	2008/England	Experience report/VII	Nurse^*^ (n=1)
S4^(27)^	Managing change in primary care: part 2	2009/England	Experience report/VII	Nurse^*^ (n=1)
S5^(28)^	Managing change in primary care: part 3	2009/England	Experience report/VII	Nurse^*^ (n=1)
S6^(29)^	Counseling and provision of long-acting reversible contraception in the US: National survey of nurse practitioners	2013/United States	Probability survey*/*VI	PHC (n=224) and women’s health (n=360) nurses
S7^(30)^	Long-acting reversible contraceptives for teenagers: Primary care recommendations	2015/United States	Literature review/VII	Not applicable
S8^(31)^	Establishing and conducting a regional, hands-on long-acting reversible contraception training Center in primary care	2018/United States	Experience report/VII	Family^*^ and pediatric nurses (n=28)
S9^(32)^	Evaluation of a pilot program for task sharing short and long-acting contraceptive methods in Burkina Faso	2020/Burkina Faso	Descriptive study of mixed methods/VI	Nurses^*^ (n=16) and registered midwives (n=8), assistant midwives (n=29), mobile health professionals (n=26), totaling 79 primary care professionals.
S10^(15)^	*Inserção de DIU por enfermeiros da Atenção Primária à Saúde*	2021/Brazil	Experience report/VII	PHC nurses (n=115)

*LoE - level of evidence; PHC - Primary Health Care;*

* *preserved the nomenclature of participants used in the studies, whose operating scenario was Primary Health Care.*

The majority (n=9; 90%) of studies were published in international scientific journals and in English. There is a predominance of experience report studies (n=6; 60%), followed by literature reviews (n=2; 20%) and quantitative or mixed methods research (n=2; 20%). Regarding the levels of evidence, it is highlighted that eight (80%) studies have level VII. As for participants, nurses (n=5; 50%), PHC nurses (n=2; 20%), family nurses (n=1; 10%), women’s health nurses (n=1; 10%) and pediatrics (n=1; 10%) were the main study subjects (n=8; 80%), since, in two (20%), as they are literature reviews, this information is disregarded (Chart 2).

The main results in relation to evidence of IUD insertion by nurses in PHC, the objectives of studies and the synthesis of the respective conclusions are found in Chart 3.

**Chart 3:** Studies selected for review according to objectives, main results and conclusion, Florianópolis, Santa Catarina, Brazil, 2022

Code	Objectives	Main results	Conclusion
S1^(24)^	To report the proposal for an IUD program that used midwives and nurses to perform the insertions.	The six-week IUD trainings involved ten nurses and ten midwives in each class. They formed four classes in 1977 and 1978, totaling 80 participants, who learned everything from contraindications to IUD insertion and removal. Each professional needed to insert 20 IUD to be considered qualified. Of the total insertions, 63% occurred in the women’s homes. More than 2,000 IUD were inserted, and there were no serious complications related to the insertions.	Nurses and midwives can safely insert IUD. Device insertion by these trained professionals increased the acceptability of the method, due to women’s bond and trust, in addition to the availability of professionals to perform the procedure at home.
S2^(25)^	To review the literature on the role of non-medical professionals as primary care providers in family planning.	In the USA, since the National Family Planning Act in 1970, nurses have received training to work in planning and women’s health. In 1967, removal, expulsion, retention, and pregnancy rates after IUD insertion in 210 women showed lower rates of expulsion and removals and higher retention rates among women who had IUD inserted by nurses (n=150) when compared to insertions by physicians (n=60). Regarding IUD insertion by physicians, obstetric nurses and rural midwives, there were no complications and significant differences between professionals. Countries such as Korea, Nigeria and Pakistan have been encouraging the training of non-physicians to insert IUD, aiming to reduce birth rates and improve access to contraception in rural areas.	Non-physicians’ ability to safely insert IUD is advocated, as they have greater acceptability and accessibility by vulnerable populations when compared to physicians. The literature reveals that there was no apparent difference in relation to IUD complications and maintenance between physicians and non-physicians. It is recommended that public services train and expand the role of nurses in family planning.
S3^(26)^	To describe the rationale for the strategies used to introduce a nurse-led service to improve LARC access and discuss the effectiveness, cost-effectiveness and training for IUD insertion.	To provide a more comprehensive and accessible family planning service, the project relies on clear evidence that nurses are competent to acquire IUD insertion skills as well as reducing costs to the practice. The Royal College of Nursing recommends that nurses insert at least five IUD and remove two IUD per year to maintain their competency.	Guidance on training and practices for IUD insertion is based on comprehensive evidence and provides a clear framework for ongoing training and practice. There is disparity in access to training, however training nurses is more economical, and they are competent in the procedure when compared to physicians.
S4^(27)^	To describe the project presentation to physician partners to introduce a nurse-led service for IUD and IUS insertion into practice and the steps required to have the clinic up and running.	A practical nurse with criteria for the role of trainer was trained by a physician to insert IUD and IUS. Practically, there was no difference between counseling and IUD insertion between physicians and nurses, except that nurses have a trained assistant. Three consultations were scheduled: the first, lasting 10 minutes, for advice, assessment and decision on the device to be inserted; in the second, lasting 20 minutes, the IUD or IUS was inserted; and after 6 weeks, a new consultation took place to check the device wires, assess bleeding and satisfaction with the method.	The success of project implementation depended in part on its application in practice, related to patients’ needs within the multidisciplinary environment.
S5^(28)^	To report the successful introduction of a nurse-led service to improve contraceptive choice, reflecting problems encountered, what was achieved and aspects of leadership in action.	The plan to develop a skill historically dominated by the medical profession was initially met with opposition. Different leadership styles were used to facilitate service implementation, and culture was identified as an important facilitator in this process. A satisfaction survey was used which revealed that patients did not attribute IUD insertion exclusively to physicians, but understood that all nurses trained in family planning already performed this procedure.	Obtaining competency in IUD/IUS insertion improved family planning skills, in addition to enabling the training of other nurses. Publication of the experience and lectures at events increased interest in the subject and inspired many nurses to train on their own.
S6^(29)^	To assess practice and training needs to prepare nurses to provide highly effective contraceptives such as IUD and implants.	Of the nurses participating in the study, 86% were trained in family planning, almost all of them working in the area of women’s health (97%). PHC nurses had greater limitations in their IUD insertion skills, with 12% of them and 72% of women’s health nurses feeling comfortable performing the procedure. IUD were most often offered by women’s health nurses, and 72% of them and 30% of PHC nurses reported including IUD in discussions with patients. The desire for training on IUD insertion was expressed by 35% of PHC nurses and 20% of women’s health nurses. The latter had more knowledge about the contraindications of the method.	Nurses provide care to vulnerable women of reproductive age in PHC settings, such as health centers as well as community family planning clinics. To address the persistent problem of unwanted pregnancy, it is essential to train nurses who provide contraceptive care to offer the most effective methods.
S7^(30)^	To discuss barriers to underuse of long-acting reversible contraceptive methods (LARC) and present an evidence-based approach to the use of these methods among adolescents in PHC.	Barriers to IUD acceptance are identified, such as lack of professional training, pain during insertion and procedure costs. Contraception is considered an essential preventive care service, and this increases the need for PHC nurses to adequately provide this service. Skills-based training in LARC methods in advanced practice nursing and continuing education programs is critical to increasing access, delivery, and utilization by adolescents in primary care.	PHC nurses are responsible for meeting the contraceptive needs of adolescents, incorporating counseling and increasing access to IUD. Furthermore, they have the potential to impact the reproductive health outcomes of adolescents. It is important to increase training in LARC methods for professionals.
S8^(31)^	To describe the experience and lessons learned developing and conducting training of PHC professionals in contraception, including LARC insertion through the Hands-on Reproductive Health Training (HaRT) Center.	A total of 28 pediatric and family nurses, 13 physicians and four physician assistants were trained. Most entered training with few speculum handling skills and limited LARC knowledge and advice. During training, professionals inserted an average of eight IUD and removed an average of two. Everyone inserted at least one IUD and 80% of them removed the device. Only 41% of trainees consider themselves competent in inserting a copper IUD and 50% in inserting a hormonal IUD. However, more than 75% of them were considered competent by the coaches. After training, 32% of participants reported that they are providing IUD insertion and 35% of them are providing device removal.	Those who entered training with basic knowledge of gynecological examination procedures and skills developed their LARC skills more quickly. It was found that family nurses have more training in speculum and gynecological examination than pediatric nurses. Services need professionals trained to provide LARC.
S9^(32)^	To assess the pilot project that aimed to share long-term family planning service tasks with the primary care team and short-term with CHW.	A total of 79 primary care providers from 26 health centers received training to provide family planning services, including IUD insertion and removal. Around 78.6% of women who had an IUD inserted were very satisfied with the method and 17.1% were satisfied. Only 12% of undesirable effects and no reports of complications after IUD insertion were revealed. Some PHC services may have been overloaded with increased access to LARC, increasing waiting times for care or the need to seek another service to obtain IUD.	The results indicate that task sharing is viable and acceptable to increase access to family planning. Before expanding the task sharing intervention, IUD insertion training is recommended for a larger number of professionals. Furthermore, attention should be paid to establishments with insufficient resources to offer LARC.
S10^(15)^	To describe the experience of nurses implementing the copper IUD insertion service in PHC in Florianópolis, Santa Catarina.	A total of 115 nurses were qualified, including permanent professionals and residents. 2,024 IUD insertions by nurses were recorded in just over three years since the service was implemented. There was a 60% increase in access and offering the method to women. In the period analyzed, nurses were responsible for the majority of IUD insertions in the city, corresponding to 58.3% of the total procedures performed.	In PHC, IUD insertion by nurses has contributed to access to the method. In this context, nurse training positively influences the qualification of care. Furthermore, the reported practice has demonstrated efficacy and safety, and goes beyond hegemonic and medical-centered models.

*IUD - intrauterine device; IUS - intrauterine system; PHC - Primary Health Care; LARC - long-acting reversible contraception; CHW - community health workers.*

From the analysis of studies^(15,24-32)^, three categories emerged: Nurse training to insert an intrauterine device; Nurses’ competency to insert an intrauterine device; and Women’s access to intrauterine devices.

### Nurse training to insert an intrauterine device

Different training experiences for IUD insertion and removal were evident [S1, S3, S4, S5, S6, S8, S9]. Note the variability of professionals who participated in training, such as nurses [S1, S9, S10], pediatric nurses [S8], family health nurses [S8], PHC nurses [S6, S10], community health nurse women [S6], resident nurses [S10], midwives [S1, S9] and physicians [S8]. Experiences show that the number of trained professionals ranged from 45 to 115 professionals [S1, S8, S9, S10]. Nurses were cited as training supervisors [S1, S4]. A study shows that nurse trainers are initially trained by a physician [S4].

Studies also address the minimum number of IUD insertions necessary for the professional to be considered qualified, with a variation of one [S8], five [S3] and 20 [S1] device insertions and also removals, which ranged between one [S8] and two [S3].

### Nurses’ competency to insert an intrauterine device

Nurses’ experiences with IUD practice reveal a significant number of device insertions in the Philippines and Brazil, where more than 2,000 [S1] and 2,024 [S10] insertions were performed, respectively. Furthermore, positive outcomes were evidenced, such as the absence of serious complications after the procedure [S1, S9], few undesirable effects [S9] and satisfaction among women [S5, S9]. Therefore, studies advocate IUD insertion by nurses, considering these professionals to be competent and qualified for the procedure [S3, S5, S7, S8, S10] as well as physicians [S2, S3, S4, S5].

### Women’s access to intrauterine devices

The studies highlight barriers in access to reproductive planning services, such as the restriction of nurses’ work [S2], the lack of professional training [S3], the medical-centered training model [S8], the overload of some health services primary care and waiting time for care [S9]. On the other hand, IUD insertion by nurses proves to be a positive strategy to solve the problem of access to IUD [S2, S7, S9, S10].

## DISCUSSION

The set of evidence analyzed allowed us to understand the factors that permeate IUD insertion by PHC nurses worldwide. Seen in these terms, the studies highlight nurse training and the competency that these professionals have to carry out IUD insertion as well as women’s access to this contraceptive method, aiming to reduce the frequent problem of unwanted pregnancy.

The findings showed records of IUD insertion by nurses over 40 years ago^(24-25)^ and, since then, different forms of training these professionals to perform the procedure have been documented^(24,27,29,31-32)^. A study carried out in 1979 described how nurses and midwives were trained to insert and remove IUD, in which each professional needed to insert 20 devices to be considered qualified^(24)^. Although the study is one of the oldest in this review, it has very current criteria on training, considering the current COFEN Resolution 690/2022, which updates the theoretical and theoretical-practical workload requirements in Brazil, expanded to a total 70 hours, establishing a minimum of 20 supervised IUD insertions for nurse certification^(17)^.

Of the studies analyzed, two^(26,30)^ reflect on the importance of training for IUD and LNG-IUS insertion being based on evidence and the skills of professionals, aiming for a clear training program and comprehensive practice. In line with this, the recent experience of training nurses in a municipality in Brazil consists of a theoretical component with a workload of 30 hours, followed by insertion training in a simulator, case discussion and consent form. After clarifying all doubts, nurses must perform at least 20 supervised IUD insertions^(33)^, which allows nurses to feel more confident during training and confident to perform the procedure after certification.

Studies have also shown that participants who began training with basic knowledge and practice in gynecological examinations more quickly developed their skills in LARC procedures, in addition to improving their learning about contraceptive counseling, contraindications, IUD insertion and removal^(24,31)^. Regarding skills in IUD insertion, research showed that PHC nurses demonstrated greater practical limitations in the procedure when compared to women’s health nurses, however 66% of these had received practical training for IUD insertion, different from those with only 12% of qualified^(29)^.

Still on the topic of training for IUD insertion and in line with the findings, a systematic review highlights that the knowledge of nurses and physicians improved from 58% to 81% after training, which was also positive for professionals’ self-confidence during the procedure^(34)^. The literature mentions self-confidence as a consequence of Advanced Practice Nursing (APN), which is based on attributes such as evidence-based practice, a high level of autonomy and advanced and broad assessment^(35)^, essential for IUD insertion by nurses. In this regard, it is noteworthy that an instrument was developed to assess nurses’ competency in inserting the device, a tool that can contribute to the qualification of these professionals^(36)^ for Advanced Practice Nursing, given that the interventions resulting from this require specialized knowledge, the ability to make complex decisions and clinical skills for practice^(37)^.

Regarding the competency of nurses to insert IUD, mentioned in the studies, it is highlighted that, upon acquiring it, nurses can positively influence the reproductive planning of adult and adolescent women, qualification of care, in addition to enabling training other nurses^(15,26,28,30)^, an aspect also considered an attribute of APN^(35)^, overcoming hegemonic models and physician-centered^(15,26,28,30)^. Research carried out in England, published in 1999, already revealed favorable evidence in this regard, highlighting that trained nurses perform efficient and safe IUD insertions, regardless of patients’ age, in addition to a better cost-benefit ratio^(13)^.

IUD insertion safety by nurses is consolidated in the outcomes of the procedure described in the studies, with emphasis on excellent satisfaction among users^(28,32)^, absence of serious complications after the procedure^(24,32)^ and low percentage of effects undesirable^(32)^. It should be noted that there was no significant difference in the outcomes of IUD insertions performed by physicians and nurses^(25-27)^, even when comparing different variables, such as removal, expulsion, retention and unwanted pregnancy^(25)^. The studies defend nurses’ competency to insert IUD and IUS^(25-26)^.

Research on the monitoring of IUD insertions by nurses and physicians at a maternity outpatient clinic showed similar results. Of the total number of IUD inserted, 61.4% were due to procedures performed by nurses; Of these, 69.9% of women did not have any complications recorded at the return visit after 30 days. After 12 months, 90% of women who had IUD inserted by nurses or physicians said they were satisfied with the device. The total continuation of the method was 85.5%, of which 53% were inserted by nurses. There was no significant difference related to complications, when compared with the insertions of physicians and nurses^(7)^.

However, some access barriers to IUD insertion were identified in the present review, such as the physician-centered training model^(25,31)^, lack of training of professionals working in the area of family planning^(26)^ and overload of care services primary care, with a consequent increase in waiting time for care^(32)^. This result is in line with two other studies^(1,9)^ that address the different barriers in access to IUD, namely: non-availability of IUD in PHC or in the municipality itself; lack of protocols for making the method available; pre-established criteria for insertion (such as medical prescription, previous exams, participation in educational groups, prior scheduling); lack of training of professionals and their knowledge of eligibility criteria; limitation of nurses’ role, making the procedure exclusive to physicians; excessive waiting time; and lack of knowledge among the population about the method.

More than half of the studies analyzed addressed access to IUD^(15,25-26,30-32)^, which indicates that this is also a prominent topic regarding device insertion by PHC nurses. Regarding the topic, nurses’ role in IUD insertion showed a greater opportunity for women to learn about LARC methods and access to device insertion. One of the Brazilian experiences reported, carried out in the city of Florianópolis, Santa Catarina, showed a 60% increase in IUD access and provision to adult women and adolescents with device insertion by nurses in PHC^(15)^. Other studies corroborate that IUD insertion by nurses is an important strategy to overcome barriers to accessing health services^(33-34)^.

Regarding access, PHC stands out as an important setting for offering IUD, as it is the gateway to health systems and one of the main reproductive planning services. In this scenario, where human resources are often a problem, especially in medical care, nurse training and performance are fundamental to qualifying contraceptive assistance and guaranteeing sexual and reproductive health care for all women^(1,38-39)^, especially those who live in situations of social vulnerability in the country or anywhere else in the world, preventing, for instance, unwanted pregnancy and abortion, with their complications and/or repercussions.

### Study limitations

The limitations of this study are mainly related to the design of most of the studies that made up the sample, characterized as experience reports, which have the lowest level of scientific evidence. Furthermore, the reduced number of studies regarding IUD insertion by nurses in PHC, when compared to other practice scenarios, limited the review sample, including in the national context.

### Contributions to nursing, health and public policies

The integrative review made it possible to group different positive experiences regarding IUD insertion by nurses in PHC and, thus, contribute to strengthening this advanced professional practice and stimulating health services to establish a training program for nurses, a strategy that can qualify contraceptive assistance and increase women’s access to IUD, which have been proven to be effective. By shedding light on knowledge production related to the topic, this review also contributes by revealing the need for robust studies with a high level of scientific evidence, such as randomized clinical trials. Furthermore, the review may support legal discussions involving device insertion and removal by nurses, giving visibility to the expansion and strengthening of public policies related to reproductive planning.

## CONCLUSIONS

Studies on IUD insertion by nurses in PHC were developed predominantly internationally, the first being published in 1979. The most recent study is Brazilian, the only one developed in the country, published in 2021 and carried out in Florianópolis. Experience report studies prevailed, classified as having a lower level of scientific evidence.

The findings, focusing on PHC, revealed three main themes: Nurse training to insert an intrauterine device; Nurses’ competency to insert an intrauterine device; and Women’s access to intrauterine devices. Different experiences of theoretical and practical training of nurses for IUD insertion were presented, with the participation of different specialties, such as pediatric, family and women’s health nurses. Trained nurses demonstrated competency in this practice, with favorable outcomes, such as the absence of serious complications after the procedure, few undesirable effects and excellent women’s satisfaction with the method. Furthermore, nurses proved to be as competent as physicians in inserting IUD. IUD insertion by nurses proved to be an important strategy to increase women’s access to this device, given the barriers to access to reproductive planning in health services.

Finally, the gaps in scientific knowledge related to IUD insertion by PHC nurses signal the need for robust research on the subject at a national and international level that uses homogeneous methodologies and representative samples, with a high level of scientific evidence, aiming to support and promote this nurses’ professional practice in PHC globally, in addition to strengthening nurses’ advanced practices in the health work process.
